# STING-induced blood-brain barrier opening combined with radiotherapy potentiates antitumor response in a high-grade glioma model

**DOI:** 10.1172/JCI198843

**Published:** 2026-02-16

**Authors:** Shashwat Tripathi, Hinda Najem, Lisa Hurley, Ruochen Du, Crismita Dmello, Heba Ali, Kathleen McCortney, Karl J. Habashy, Peng Zhang, Craig M. Horbinski, Lara Leoni, Ryan J. Avery, Rimas V. Lukas, Timothy L. Sita, David R. Raleigh, Sean Sachdev, Roger Stupp, Maciej S. Lesniak, David M. Ashley, Daniele Procissi, Michael A. Curran, Irina Balyasnikova, Amy B. Heimberger

**Affiliations:** 1 Department of Neurological Surgery,; 2 Malnati Brain Tumor Institute of the Robert H. Lurie Comprehensive Cancer Center,; 3 Department of Radiology,; 4 Department of Neurology, and; 5 Department of Radiation Oncology, Feinberg School of Medicine, Northwestern University, Chicago, Illinois, USA.; 6 Department of Radiation Oncology, Neurological Surgery, and Pathology, University of California, San Francisco, San Francisco, California, USA.; 7 Department of Medicine, Duke University School of Medicine, Durham, North Carolina, USA.; 8 Department of Immunology, The University of Texas MD Anderson Cancer Center, Houston, Texas, USA.

**Keywords:** Immunology, Oncology, Vascular biology, Brain cancer, Cancer immunotherapy, Therapeutics

## Abstract

Radiation therapy (RT) is the standard of care for glioblastoma but is not curative. Triggering the cGAS/stimulator of interferon genes (STING) pathway with potent agonists, such as 8803, exerts activity across high-grade glioma preclinical models. To determine if the combination of 8803 with RT warrants consideration in the up-front treatment setting and to clarify the underlying mechanisms of therapeutic activity, C57BL/6J mice harboring intracerebral CT-2A or QPP8v gliomas were treated with RT, intratumoral 8803, or both. The treatment with the combination resulted in 80% long-term survival in the CT-2A model but not in the radiation-resistant QPP8v model. This therapeutic effect was maintained in *Sting*^–/–^ CT-2A cells, highlighting the direct role of the immune system in mediating the survival benefit. Single-cell RNA-Seq identified increased nitric oxide synthase 2 (*Nos2*) in inflammatory tumor-associated macrophages; however, the therapeutic effect was maintained in *Nos2^–/–^* mice. Additionally, 8803 reprogrammed the blood-brain barrier (BBB) by altering the Pecam and Cd147 pathways in endothelial cells; intracranial injection of 8803 induced bihemispheric BBB opening for up to 24 hours. Sting activation was visualized longitudinally using 3’-deoxy-3’-[^18^F]-fluorothymidine ([^18^F]-FLT) PET, which peaked 72–96 hours after 8803 administration. In summary, 8803 combined with RT triggers distinctive antiglioma immune reactivity, facilitates BBB opening, and warrants consideration for up-front clinical trials in glioblastoma, where treatment effects can be monitored using [^18^F]-FLT PET imaging.

## Abstract 2

This study demonstrates the therapeutic benefit of adding the STING agonist 8803 to radiation in preclinical models of glioma that would justify a clinical trial in the newly diagnosed patient setting of glioblastoma. The combination reprograms the glioma tumor microenvironment, and 8803 induces the opening of the blood-brain barrier. Since only a few agents achieve sufficient concentrations within the brain to mediate therapeutic activity for gliomas from the current compendium, this novel observation provides a new and significant avenue for future investigation for combinatorial strategies. Activation of the STING pathway can be visualized with PET, that will be incorporated into the pending clinical trial

## Introduction

The cGAS/stimulator of interferon genes (STING) pathway is a widely expressed sensor of cellular stress. STING is activated by cyclic dinucleotides that are shed by dying tumor cells. STING agonists are of therapeutic interest because they induce chemokine production, leading to T cell trafficking to the tumor and pro-inflammatory interferon (IFN) responses that mediate immune cytotoxic effector activation. The activation of the STING pathway also reprograms myeloid cells to a pro-inflammatory and antitumor M1 phenotype. STING agonists are scalable, inexpensive, and easy to generate as a clinical-grade product (1). The high-potency STING agonist, 8803, has demonstrated antitumor activity in a wide variety of preclinical high-grade glioma models, including those resistant to immune checkpoint therapy (2), and in dogs with spontaneously arising high-grade gliomas (2). In glioblastoma, the STING gene promoter is highly methylated regardless of isocitrate dehydrogenase or *O*-6-methylguanine-DNA methyltransferase (MGMT) status (3). Collectively, STING pathway activation and therapeutic activity have been attributed to the immune-mediated antitumor cytotoxicity.

Radiation therapy (RT) triggers the cytosolic entry of mitochondrial dsDNA through oxidative damage and modifications, resulting in cGAS activation (4). Radiation-induced oxidative damage results in direct tumor cell death and secondary immune-mediated cytotoxicity. STING also regulates the transcriptional program that controls the generation of reactive oxygen species (ROS) (5). STING loss alters ROS homeostasis to reduce DNA damage associated with therapeutic resistance. Pharmacological activation of STING expression can enhance the effects of ionizing radiation in vivo in the preclinical treatment of squamous and pharyngeal cancers (5). However, this combination has not been evaluated in gliomas, where STING expression is epigenetically silenced in the tumor cells. Furthermore, the STING pathway activation can be countered by the radiation-induced nuclear factor erythroid 2-related factor 2 (NRF2) pathway. Others have shown that RT can upregulate YTH domain family protein 1 (YTHDF1) in dendritic cells and trigger STING degradation by increasing lysosomal cathepsins, thereby reducing IFN production (6). Hemeoxygenase-1 (HO-1) can also impair the efficacy of RT through the inhibition of the cGAS/STING pathway (7). Since RT is the standard of care for newly diagnosed glioblastoma, preclinical assessments of the combination of RT and 8803 would inform clinical trial indications in MGMT-unmethylated gliomas.

A challenge in the clinical translation of immunotherapeutic strategies is the lack of noninvasive indicators of immune activation in the tumor microenvironment (TME). It has been reported that the nucleoside analog and PET probe 3′-deoxy-3′-[^18^F]-fluorothymidine ([^18^F]-FLT) can visualize IFN signaling–induced alterations in the TME via PET/CT (8). The authors found that IFN signaling augments pancreatic cancer cell nucleotide metabolism by inducing transcription of metabolism-associated genes, such as thymidine phosphorylase (TYMP). TYMP catalyzes the first step in the catabolism of thymidine, and this upregulation depletes intracellular thymidine, which increases the intratumoral accumulation of [^18^F]-FLT. Accordingly, IFN treatment increases [^18^F]-FLT uptake in cancer cells in the presence of thymidine, and this effect depends on TYMP expression. In the current study, we aimed to determine if PET imaging could be applied to detect an increase in tracer uptake in high-grade gliomas and serve as a radiographic biomarker of STING pathway activation for monitoring STING-induced activation in glioblastoma.

## Results

### 8803 exerts an additive therapeutic benefit to radiation.

**Figure 1:**
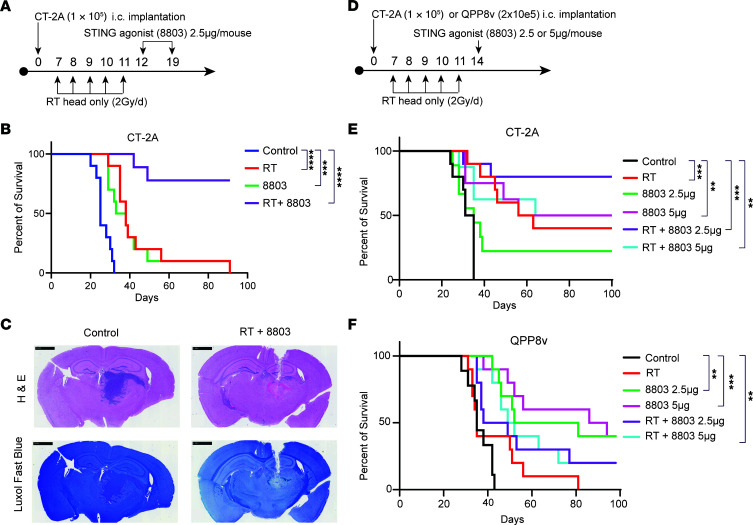
Therapeutic effect of 8803 in combination with RT. (**A**) Treatment of C57BL/6J mice with intracerebral (i.c.) CT-2A implantation. (**B**) Mice treated with RT + 8803 (*n* = 10, undefined MS) had significantly prolonged survival vs. vehicle control mice (*n* = 10; MS = 25d; *P* < 0.0001). RT (*n* = 10; MS = 38d) vs. control (*P* = 0.0001); 8803 (*n* = 10; MS = 36d) vs. control (*P* = 0.0005). 8803 vs. RT (*P* = 0.808). Log-rank. (**C**) Representative coronal images of H&E- and Luxol Fast Blue–stained mouse brains. Decreased staining of Luxol Fast Blue corresponds to region of regressed tumor. Scale bars: 1 mm. Magnification: 2.33×. (**D**) Schema showing schedule modification for clinical trial modeling. (**E**) Mice bearing orthotopic CT-2A treated with RT + 8803 at 2.5 µg dose (*n* = 10; undefined MS) showed significantly prolonged survival vs. control mice (*n* = 10; MS = 33d; *P* = 0.0002). RT + 8803 at 5 µg dose (*n* = 10; MS = 74) vs. control (*P* = 0.0079). RT (*n* = 10; MS = 59.5d) vs. control (*P* = 0.0002). 8803 at 2.5 µg (*n* = 10; MS = 35) vs. control (*P* = 0.165). 8803 at 5 µg (*n* = 10; MS = 70d) vs. control (*P* = 0.0046). 8803 at 2.5 µg vs. RT (*P* = 0.098). 8803 at 5 µg vs. RT (*P* = 0.7915). Log-rank. (**F**) Survival rate of mice bearing orthotopic radiation-resistant QPP8v treated with RT has a similar MS (*n* = 10; MS = 35d) relative to PBS controls (*n* = 9; MS = 35d). Treatment with 8803 at 2.5 µg (*n* = 10; MS = 61d) or 5.0 µg dose (*n* = 10; MS = undefined) on day 14 significantly prolonged survival relative to PBS control mice (*P* < 0.0001). However, there was no additional therapeutic benefit of RT with 8803 at 2.5 (*n* = 10; MS = 51d; *P* = 0.499) or 5 µg dose (*n* = 10; MS = 57.5d; *P* = 0.348) when compared with 8803 2.5 µg and 5 µg monotherapies, respectively. RT + 8803 2.5 µg vs. control (*P* = 0.0071). RT + 8803 5 µg vs. control (*P* = 0.0002). Log-rank. ** < 0.01; *** < 0.001; **** < 0.0001.

To evaluate the therapeutic impact of adding 8803 to RT, we administered a standard, daily, 2 Gy dose for 5 days. Since 8803 induces the influx of effector T cell responses into the TME, the combinatorial schedule was sequenced to minimize radiation-induced T cell apoptosis. Immune-competent mice with established CT-2A glioblastoma were treated with monotherapy or in combination. RT was initiated on day 7, followed by administration of 8803 on days 12 and 19 (Figure 1A). Male and female mice were randomized into treatment groups (*n* = 10 mice/group). A lower dose (2.5 μg/mouse) of 8803 was administered intratumorally to determine if RT had a synergistic effect. Control mice had a median survival (MS) time of 25 days, 8803-treated mice had an MS of 36 days (*P* = 0.005), RT provided an MS of 38 days (*P* < 0.0001), and the MS in the combination group was undefined (*P* < 0.0001) (Figure 1B). There was no difference in survival between male and female mice. To determine if this was an additive or a synergistic effect, a CISNE assessment (9) was made, which showed significance with STING [Pr(>|z|)=0.0001] and RT [Pr(>|z|)=0.00006] monotherapies, but not with the combination [Pr(>|z|)=0.68], indicating that the combination has an additive effect rather than synergistic.

### CNS toxicity assessments with 8803 and radiation treatment.

Mice were monitored daily to assess whether the combination of 8803 and RT induces toxicity. No neurological symptoms were noted until the final survival endpoint. The neuroaxis was stained for myelin with Luxol Fast Blue when the mice succumbed to the tumor. On histologic examination, there was no evidence of demyelination (*n* = 3 per group) (Figure 1C), as reviewed by a board-certified neuropathologist. Additionally, perivascular lymphocytic infiltrates were not observed on hematoxylin and eosin staining, suggesting the lack of induction of autoimmunity. Neuropathologic analysis failed to identify perivascular fibrosis or persistent demyelination, consistent with the lack of subclinical autoimmunity occurring at any point during the animal’s lifespan. These results are consistent with more extensive toxicity studies on 8803 monotherapy (10).

### Clinical trial modeling of a combinatorial schedule demonstrates therapeutic responses.

Since treatment with 8803 requires direct intratumor administration, it would be challenging to have a patient complete radiation on the same day as the stereotactic surgical delivery of 8803. Additionally, the cost, risks of complications, and burden on the patients to undergo 2 stereotactic procedures in 1 week would limit accrual. As such, we repeated the experiment, waiting 72 hours after radiation completion, to treat the mice with a single dose of 8803. Mice were randomized into the following treatment groups (*n* = 10 mice/group): control, RT, 8803 (2.5 μg), 8803 (5 μg), RT + 8803 (2.5 μg), and RT + 8803 (5 μg) (Figure 1D). A single dose of 8803 at 2.5 μg + RT showed a significant survival benefit (MS undefined, *P* = 0.0002 vs. control, 80% survival rate) relative to a dose of 5 μg + RT (MS of 74 days, *P* = 0.0079 vs. control, 50% survival rate) (Figure 1E). This reduced therapeutic benefit was not due to RT-induced NRF2 expression (Supplemental Figure 1; supplemental material available online with this article; https://doi.org/10.1172/JCI198843DS1). Control mice had an MS of 33 days, 8803-treated mice (2.5 μg) had an MS of 35 days (*P* = 0.165), 8803-treated mice (5 μg) had an MS of 70 days (*P* = 0.0046), and RT-treated mice had an MS of 59.5 days (*P* = 0.0002). Differential MS has been previously noted between experiments in the CT-2A model treated with RT (11). Next, using the same dose and schedule (Figure 1D), this combinatorial strategy was evaluated in the QPP8v radiation-resistant glioma model, in which both control and RT-treated mice had an MS of 35 days. In this model, RT did not confer additional therapeutic benefit to 8803, as the combination had an MS of 43.5 or 50.5 days (2.5 or 5 μg, respectively). In contrast, 8803 dosed at 2.5 or 5 μg had an MS of 66.5 and 90 days, respectively (Figure 1F).

### RT and 8803 immunologically reprogram the glioma TME to a pro-inflammatory state.

**Figure 2:**
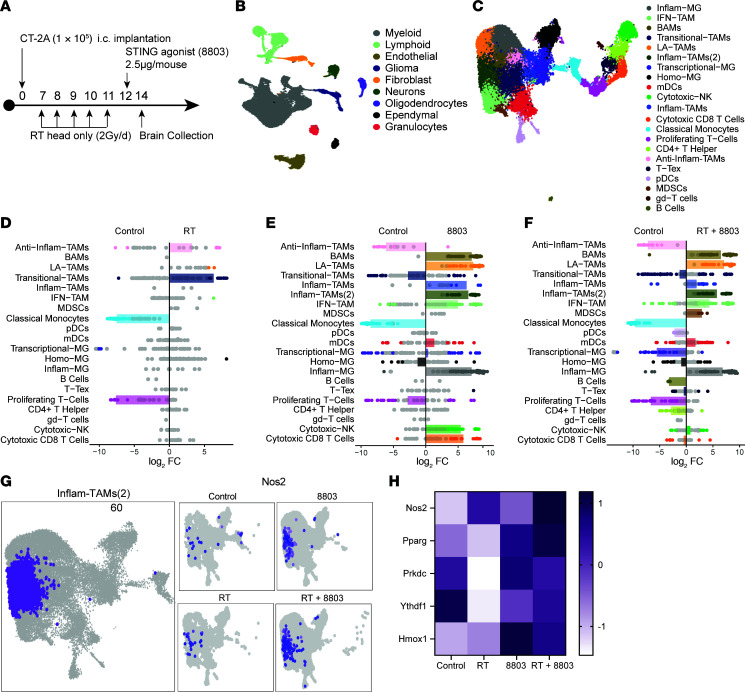
Ex vivo correlative analysis of the TME using scRNA sequencing and multiplex immunofluorescence. (**A**) Schema demonstrates that the therapeutic window analysis was conducted on brain samples collected 48 hours after 8803 administration. Brain samples were either freshly processed for scRNA-Seq analysis or formalin-fixed and paraffin-embedded for spatial multiplex immunofluorescence staining. (**B**) Uniform manifold approximation and projection (UMAP) plot showing the cellular composition of the CT-2A TME based on gene signatures of the scRNA-Seq dataset. (**C**) UMAP plot of the detailed clustering of the various immune populations present within the TME based on the RNA signatures. BAM, border-associated macrophage; TAM, tumor-associated macrophage. (**D**–**F**) Strip plots showing the differential abundance of immune cell types in CT-2A–bearing C57BL/6 mice based on treatment compared with control, log_2_(FC). The dot is colored when the *P* value is less than 0.05. The box plot represents the mean of significant points for immune populations with more than 3 significant clusters. Cell types with *P* ≥ 0.05 are colored in gray. *P* value calculated using negative binomial generalized linear model with weighted false discovery rate multiple correction. (**G**) t-Distributed stochastic neighbor embedding (t-SNE) plots show the cluster of Inflam-TAMs(2) (left panel) and the *Nos2* gene expression within this population as a function of treatment condition (right smaller panels). (**H**) Heatmap of differential expression of *Nos2*, *Pparg*, *Prkdc*, *Ythdf1*, and *Hmox1* genes based on treatment conditions: control, RT, 8803, and RT + 8803.

Since RT is the standard of care in glioblastoma, with most patients demonstrating clinical benefit, the radiation-sensitive CT-2A model was selected to characterize the immunological responses within the TME. During the therapeutic window of treatment, control and radiation-, 8803-, or combination-treated mice were terminated on day 14 for analysis of tissue by single-cell RNA (scRNA) sequencing (Figure 2A). The scRNA-sequencing data showed that myeloid cells were the dominant cellular population in the glioma TME (Figure 2, B and C). An unbiased differential expression of gene analysis of upregulated genes (log FC ≥ 1.0) within the myeloid cells between the treatment conditions revealed 125 genes that were uniquely upregulated in the RT + 8803 combination therapy group relative to monotherapy or PBS control conditions. The top upregulated genes included those that amplify interferon responses (e.g., *Trim21*, *Parp9*, and *Stat2*) or that modulate immune reactivity, including in microglia (e.g., *Pou3f1*, *Alox5ap*, *Dram2*, and *Npc1*). When the differentially expressed genes (DEGs) were analyzed based on genes that were upregulated in the combination treatment group but were downregulated with RT, *S1pr2* (expressed on neurons and vascular cells that promote migration and growth of injured neurons and vasculature; ref. 12), *Ifit3b* (interferon induction), and *Ifi214* (interferon activation) were the top-ranked genes induced. Top genes that were downregulated in the combination but upregulated with RT included *Hbegf* (*Egfr* signaling), *Il1a* (inflammation), *Vegfa* (angiogenesis), *Igf1* (growth factor), *Lag3* (immune checkpoint), *Tnfaip2* (TNF-α), *Ccl17* (Treg chemokine), and *Vcam1* (endothelial cell–enhanced immune cell adhesion), indicating the combination supports a less immune-suppressed TME.

### Induced NOS is associated with the combinatorial activity of RT and 8803.

Subclustering of the immune components was based on previously described strategies (13–16). RT increased innate immunity within the TME as reflected by the expansion of transitional and antiinflammatory TAMs (Figure 2D). Consistent with our prior profiling (10), 8803 induced innate and adaptive inflammatory responses, including lipid-associated (LA-), inflammatory (Inflam-), and IFN-TAMs; inflammatory microglia (MG); cytotoxic NK cells; and CD8^+^ T cells in the glioma TME (Figure 2E). Expansion of BAMs that limit brain inflammatory responses (17) was also noted (Figure 2E). The immune composition of the RT + 8803–treated TME was like 8803-treated gliomas (Figure 2F) but without a statistical enrichment of cytotoxic T cells, likely because of kinetic differences between the 2 treatment groups. The Inflam-TAM(2) population was noted to be enriched in the TME of mice treated with 8803 or RT + 8803 but not in the RT or control group (Figure 2, D–F). Many of these immunological effects are likely dominated by 8803 administration in the RT + 8803–treated TME (Supplemental Figure 2). Differences in the frequency of cytotoxic populations in the RT + 8803 versus 8803 are likely influenced by the temporal kinetics of scRNA-sequencing analysis relative to the initial induction times of the treatments. The top-upregulated gene in Inflam-TAM(2) was *Nos2* (Figure 2, G and H), which encodes nitric oxide synthase that catalyzes the formation of nitric oxide — a reactive free radical with antitumor activity. NO can modulate tumor DNA repair mechanisms through the upregulation of poly(ADP-ribose) polymerase (PARP; *Pparg* gene) or DNA-dependent protein kinase (DNA-PK; *Prkdc* gene). Notably, *Pparg* expression was highest in the RT + 8803–treated group (Figure 2H).

RT can downregulate the STING pathway via Ythdf1 or Hmox1 (gene for HO-1). To clarify whether these genes contribute to the therapeutic resistance of RT + STING in gliomas, we quantified their expression across treatment groups. There was no significant difference in *Ythdf1* across RT treatments in the glioma models. This may be due to differences between cancer lineages, as the prior study was in melanoma (6). Alternatively, this difference may be attributed to the extent of dendritic cell infiltration in which *Ythdf1* is expressed. Dendritic cell infiltration was typically low in gliomas (Figure 2, D–F). Instead, gliomas are enriched with macrophages and microglia (18). In contrast, *Hmox1* expression was upregulated in gliomas treated with 8803 versus control (log FC > 2.26), RT + 8803 versus control (log FC > 1.74), and RT + 8803 versus RT (log FC > 1.31), but not in RT versus control conditions, suggesting that activation of the STING pathway induces autocrine regulation through the Hmox1 pathway.

### 8803 reprograms intracellular interactions, including pathways that are associated with the blood-brain barrier.

**Figure 3:**
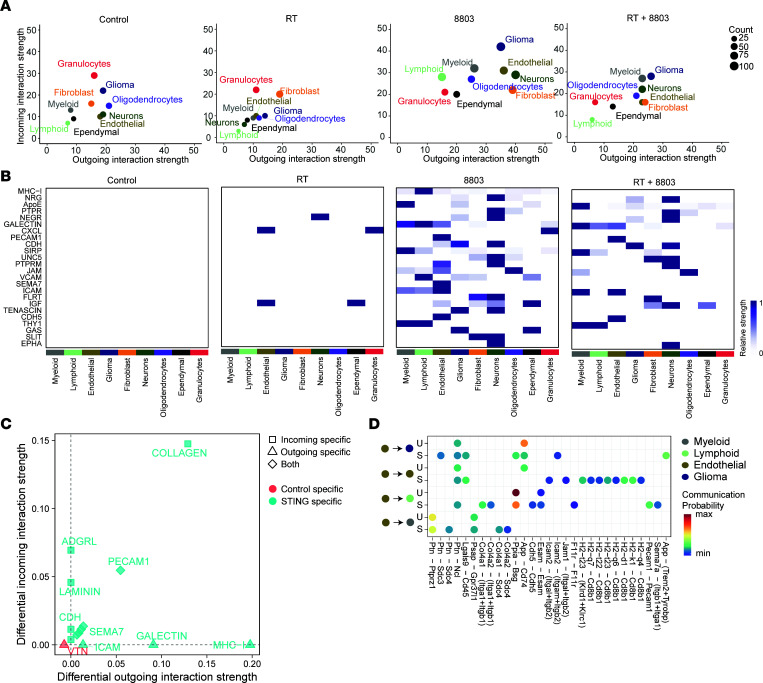
8803 reprograms intracellular interactions, including pathways that are associated with the BBB. (**A**) CellChat analysis of scRNA sequencing of immunocompetent C57BL/6J mice with intracerebral implantation of CT-2A glioma cells treated with RT (2 Gy/d) for 5 days starting on day 7 and/or with 8803 (2.5 μg/mouse) on day 12 (24 hours after the last RT) after glioma cell implantation. Cell lineages were annotated as shown in Figure 2B, and interaction strengths were measured. (**B**) Heatmaps showing the significantly utilized pathways upregulated with the designated treatment and associated with cell lineage. Only pathways with *P* < 0.05 are selected by the algorithm within CellChat. (**C**) Analysis of significant pathways within endothelial cells (as either source/outgoing or target/incoming) between STING versus PBS control animals. (**D**) Differential expression analysis (DEA) of predicted ligand-receptor analysis showing up- and downregulated L-R pairs in STING versus PBS control. Dots represent L-R pairs with predicted *P* < 0.01 from Wilcoxon’s rank-sum test. The color legend displays the scaled communication probability. Endothelial cells were considered the source (ligand), and targets were myeloid, lymphoid, glioma, and other endothelial cells. U, control; S, STING/8803-treated.

To elucidate whether there were changes in cell-to-cell interactions as a function of treatment conditions, we used CellChat to interrogate the strength of incoming and outgoing interactions based on cell lineage (Figure 3A). Monotherapeutic RT did not significantly alter the overall interaction strength among most cell populations; however, reprogramming of cellular interactions was most profound with 8803 treatment. Relative to baseline control conditions, treatment with either 8803 or the combination of RT increased intercellular communication within glioma, myeloid, neuronal, and endothelial cells. Examination of significantly utilized signaling pathways unique to 8803 or the combination included known downstream STING pathway targets such as MHC but also blood-brain barrier (BBB) regulation pathways including ICAM, CDH5, JAM, SEMA7, and PECAM, which are notable in endothelial cells (Figure 3B). Further interrogation of endothelial cell–specific pathways showed similar STING-mediated increases in PECAM1, CDH, ICAM, and SEMA7 (Figure 3C). PECAM signaling is involved in regulating BBB permeability, restoration of BBB integrity, and paracellular T cell diapedesis (19) and was upregulated with 8803 in the endothelial cells, likely enabling the increased glioma immune infiltration. Characterization of the ligand-receptor (L-R) interactions indicated that endothelial-to-endothelial cell communication was the highest upon treatment with the STING agonist 8803 (Figure 3D). Using differential expression analysis of L-R signaling, 8803, as expected, enhanced endothelial-lymphoid interactions through HLA (H2) and CD8^+^ T cell interactions (Figure 3D). Additionally, the Ppia-Bsg/CD147 L-R pair was downregulated upon treatment with STING (Figure 3D). CD147 is expressed on endothelial cells and is responsible for BBB maintenance (20). Cumulatively, these bioinformatic analyses suggest that 8803 may have a significant role in modulating the BBB.

### 8803 opens the BBB.

**Figure 4:**
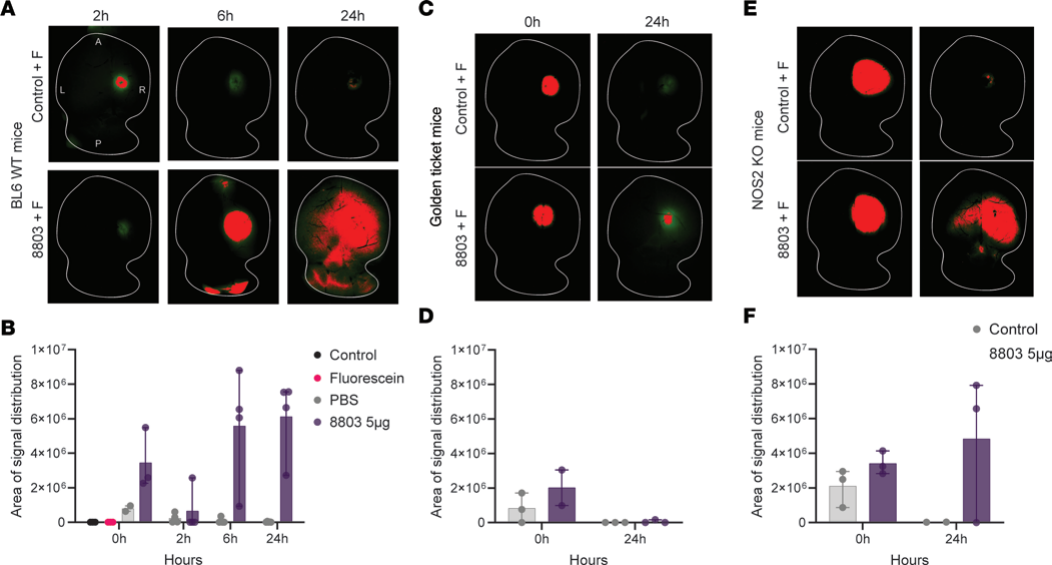
8803 triggers BBB opening. (**A**) Representative explanted whole brain mount images from the experimental treatment groups. After fluorescein dye administration, mouse brains were collected and imaged using the Nikon epifluorescence microscope at 4× original magnification. Quantitative data were generated based on intensity thresholds, eliminating noise and then harmonizing across all images. Positive-intensity data points (pixels) are shown in red, and the baseline fluorescent expression is shown in green. The brain outline is illustrated with the white line. Brightness color adjustments were made to detect baseline green fluorescence of the brains for illustrative purposes, which does not alter the quantitative data generated based on the intensity thresholds harmonized across all images. (**B**) Summarized quantification of the fluorescent signal area of distribution between experimental groups (*n* = 2–4). (**C**) Representative images of explanted whole brain mounts from the experimental groups collected at baseline and 24 hours after intracerebral administration of either PBS or 8803 (5 μg) in the golden ticket background mice. (**D**) Summarized quantification of the fluorescent signal area of distribution between experimental groups, as shown in **C**. (**E**) Representative images of explanted whole brain mounts from the experimental groups collected at baseline and 24 hours after either PBS or 8803 (5 μg) administration in the NOS2-KO mice. (**F**) Summarized quantification of the fluorescent signal area of distribution between experimental groups as shown in **E**.

The CNS is an immune-privileged organ in which endothelial and glial cells block entry, thereby limiting the effective penetration of therapeutics through the BBB. STING is expressed in the endothelial cells of brain tumors (10), but its role in modulating the BBB has not been previously evaluated to our knowledge. Inducible NO synthase (iNOS) directly affects cerebrovascular tone, endothelial cell permeability, and endothelial junctional integrity (21–23). Since Nos2 is upregulated in CT-2A mice treated with RT, 8803, or the combination relative to the control (Figure 2, G and H), the opening of the BBB in non-tumor-bearing mice was assessed across these treatment groups: 1) untreated; 2) intravenously administered fluorescent dye alone; 3) intraparenchymally injected PBS and fluorescent dye; 4) intraparenchymally injected 8803 and fluorescent dye; 5) RT + fluorescent dye; 6) PBS + RT + fluorescent dye, and 7) RT + 8803 + fluorescent dye and then terminated 24 hours after RT or at 2, 6, or 24 hours after 8803 or PBS treatment. The fluorescent signal in the brains was similar in the untreated, fluorescent dye alone, and RT + fluorescent dye groups, indicating no significant difference in BBB breakdown (Supplemental Figure 3, A and B). As expected, injecting the brain with PBS induced a localized transient signal of fluorescent dye leakage that peaked at 2 hours and then dissipated (Figure 4A). In contrast, the localized injection of 8803 induced a greater signal that peaked at 6 hours and persisted for 24 hours posttreatment (Figure 4, A and B). Notably, this increased fluorescence was detected in regions of the brain that were not adjacent to the injection site, such as the contralateral hemisphere and cerebellum (Supplemental Figure 3C). RT at a single 2 Gy dose in combination with 8803 did not substantially increase the BBB opening in comparison with monotherapy 8803 (Figure 4A and Supplemental Figure 3B). When the brains were sectioned coronally through the area of 8803 injection at 24 hours, this fluorescent signal was detected robustly throughout the entire right-sided hemisphere and even into the contralateral hemisphere (Supplemental Figure 3, C and D). To define the underlying mechanism of the BBB opening, *Nos2*-knockout (NOS2-KO) mice or golden ticket background mice were treated with either focal PBS or 8803 and fluorescent dye and then analyzed at 0 and 24 hours. The signal was completely lost in the golden ticket STING-KO background mice (Figure 4, C and D), and there was a slight decrease in the fluorescent signal intensity in the brains of the NOS2-KO mice (Figure 4, E and F), confirming that STING plays a pivotal role in BBB permeability and that iNOS is a minor contributor at this time point. Dexamethasone, routinely used during the treatment of gliomas, did not alter 8803-mediated BBB opening (Supplemental Figure 3E). These data indicate that, in addition to the known role of STING agonists in triggering immune cell chemokines, the STING pathway also plays a key role in BBB breakdown, likely contributing to increased immune infiltration into the glioma TME.

### RT and 8803 exert therapeutic efficacy when STING is silenced in the glioma cell.

**Figure 5:**
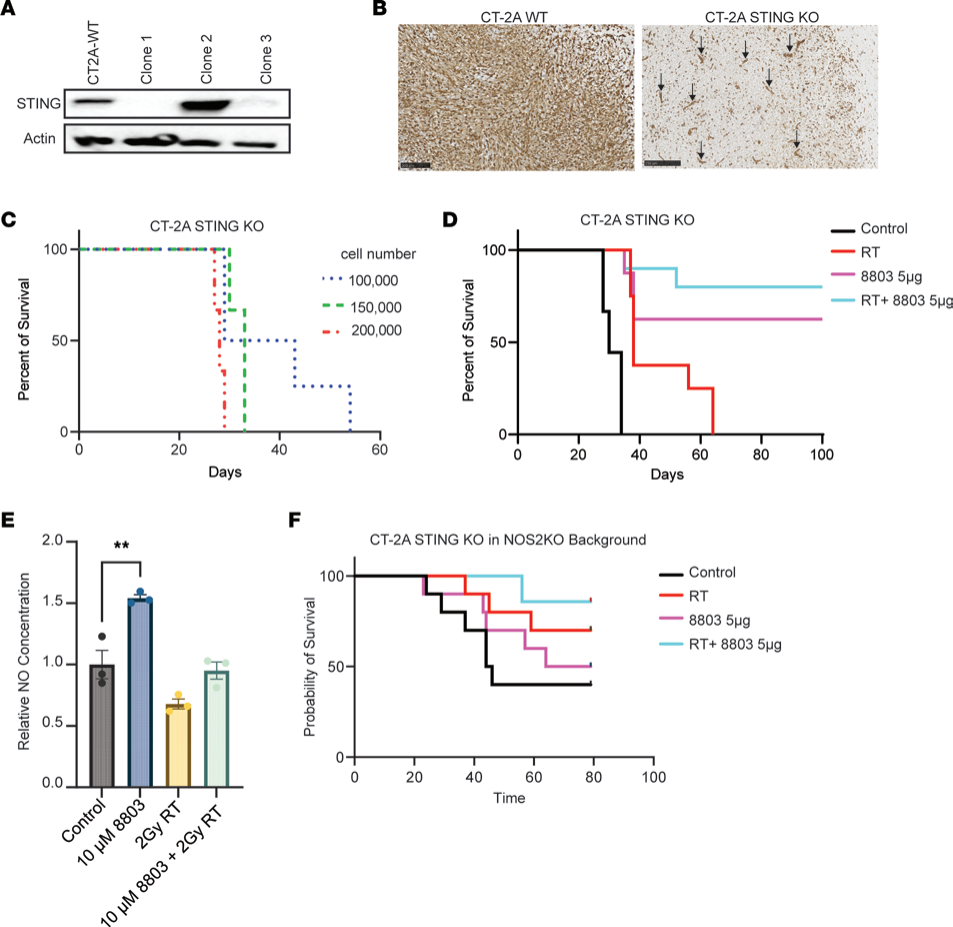
Treatment effect of 8803 in combination with radiation in STING-KO CT-2A glioma model. (**A**) Western blot analysis of the STING expression in the WT CT-2A glioma cell line and STING-KO CRISPR clones. Clone 3 showed >80% reduction in STING expression and was used for the in vivo studies. (**B**) Immunohistochemistry staining of STING expression in WT CT-2A and STING-KO cells implanted into C57BL/6J mice (black arrows designate STING^+^ blood vessels). Scale bar is 100 µm (left) and 250 µm (right). (**C**) Survival of mice implanted with CT-2A STING-KO glioma cells. (**D**) Mice with STING-KO CT-2A gliomas were treated with RT (2 Gy/d) for 5 days starting on day 7 and then with 8803 (5 μg) on day 14. Survival of mice treated with RT + 8803 (*n* = 10, undefined MS) was significantly prolonged relative to control mice (*n* = 10; MS = 30d; *P* < 0.0001). RT (*n* = 10, MS = 38d) vs. control (*P* < 0.0001). 8803 (*n* = 10, undefined MS) vs. control (*P* < 0.0001). Log-rank. Control mice had an MS time of 30 days, and the 8803-treated mice had an MS that was undefined (*P* < 0.0001). (**E**) NO production from RAW 264.7 in triplicate, normalized to control. **P < 0.01. Analyzed using 1-way ANOVA with Tukey’s multiple comparisons test. (**F**) NOS2-KO mice with STING-KO CT-2A gliomas were treated with RT (2 Gy/d) for 5 days starting on day 7 and then with 8803 (5 μg) on day 14. Survival of the mice treated with RT + 8803 (*n* = 10, undefined MS) was significantly prolonged relative to control mice (*n* = 10; MS = 45d; *P* < 0.05). RT (*n* = 10, MS = undefined) vs. control (*P* < 0.13). 8803 (*n* = 10, 72d MS) vs. control (*P* < 0.54). Log-rank.

Because CpG promoter methylation does not occur in most immune-competent murine glioma cell lines, a murine glioma cell line that more accurately reflects this scenario was created using CRISPR gene editing to knock out STING in CT-2A high-grade glioma cells to evaluate the efficacy of the combination of 8803 and RT. A CRISPR KO of STING in CT-2A glioma cells was created that showed more than 80% reduction in STING expression (clone 3) compared with the parental WT CT-2A (Figure 5A). The absence of STING expression in the tumor cells in vivo was verified using immunohistochemistry (IHC) of the brains implanted with CT-2A (Figure 5B). Notably, although STING expression was absent in the glioma tumor cells, its expression was maintained in the endothelial cells and the infiltrating macrophages. *Sting*-KO CT-2A cells showed consistent tumorigenic growth in vivo (Figure 5C) with similar cell requirements (at 1 × 10^4^ cells) as WT CT-2A. Furthermore, the outcomes of mice with i.c. *Sting*-KO CT-2A were similar to those of WT CT-2A. More specifically, in the *Sting*-KO CT-2A model, control mice had an MS time of 30 days, 8803-treated mice had an MS that was undefined (*P* < 0.0001), RT had an MS of 38 days (*P* < 0.0001), and the combination was undefined (*P* < 0.0001) (Figure 5D). These outcomes highlight that the therapeutic efficacy of RT + 8803 is independent of tumor cells’ STING expression in the murine model.

### 8803 induces NO in macrophages but is not the primary mediator of therapeutic activity.

To ascertain if RT, 8803, or RT + 8803 induces NO, the RAW 264.7 macrophage cell line was treated with 2 Gy of RT, 8803, or the combination. NO was measured in triplicate 24 hours after RT and 12 hours after 8803 administration. NO expression after treatment was highest in the 8803 monotherapy group (Figure 5E), indicating that the iNOS-mediated effects through macrophages are primarily induced with 8803. To evaluate the therapeutic contribution of NO induction to the combinatorial effect, mice homozygous for the *Nos2^tm1Lau^* targeted mutation (also known as iNOS^–/–^) were implanted with STING-KO CT-2A and then randomized to treatment with monotherapy or the combination. RT was initiated on day 7, followed by administration of 8803 (5 μg) on day 14. Control mice had an MS time of 45 days, 8803-treated mice had an MS of 72 days (*P* = 0.54 relative to control), RT provided an undefined MS (*P* = 0.13 relative to control), and the combination had an undefined MS (*P* < 0.05, relative to the control) (Figure 5F). Treatment of *Sting*-KO CT-2A gliomas with the RT + 8803 combination in the C57BL/6J (Figure 5D) and NOS2-KO backgrounds (Figure 5F) had an undefined MS, indicating that NO is a minor contributor to the combinatorial therapeutic response. The increased survival of *Sting*-KO CT-2A gliomas implanted in the *Nos2*-KO mice relative to WT is consistent with other tumor models implanted in this background (24).

### 8803 in vivo activity can be assessed using [^18^F]-FLT PET.

**Figure 6:**
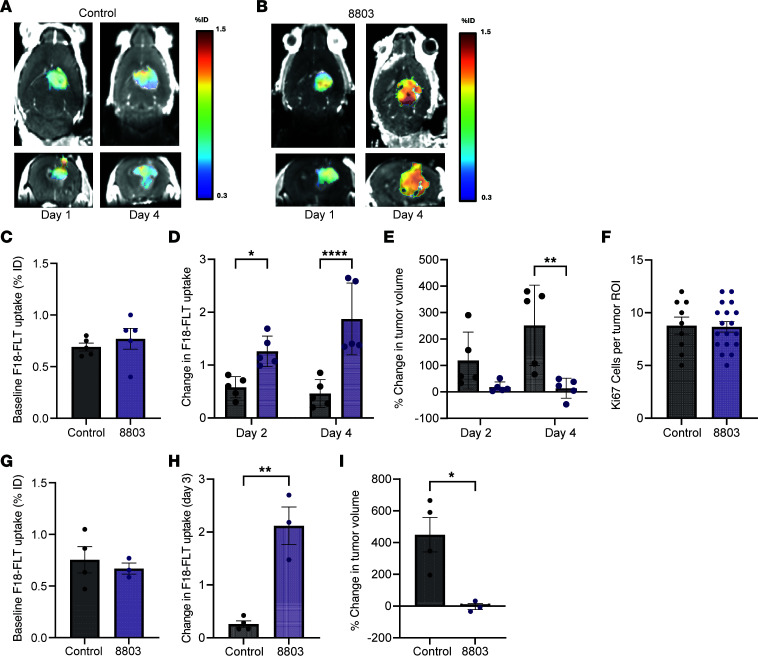
8803 in vivo activity determined using [^18^F]-FLT PET imaging. Representative MRI and [^18^F]-FLT PET merged axial and coronal images of mouse brains implanted with CT-2A gliomas analyzed at baseline (time 0) and 96 hours (4 days) in control (**A**) and after 8803 treatment (**B**). %ID, percentage of injected dose. (**C**) Baseline [^18^F]-FLT uptake in control (*n* = 5) and 8803-treated CT-2A glioma (*n* = 5). Error bars show the standard error of the mean. There was no significant difference. (**D**) Change in [^18^F]-FLT uptake at 48 and 96 hours in control versus 8803 animals. * < 0.05; **** < 0.0001. (**E**) Percentage change in tumor volume as measured on MRI between baseline and 48 or 96 hours. ** < 0.01. (**F**) Quantification of Ki-67^+^ proliferating tumor cells between control (*N* = 3) versus 8803 (*n* = 6). A total of 3 ROIs per tumor were chosen. (**G**) Baseline [^18^F]-FLT uptake in control (*n* = 4) and 8803-treated CT-2A STING-KO glioma (*n* = 3). Error bars show the standard error of the mean. (**H**) Change in [^18^F]-FLT uptake at 72 hours in control versus 8803 animals. ** < 0.01. (**I**) Percentage change in tumor volume as measured on MRI between baseline and 72 hours. * < 0.05. Panels **C**–**E** and **G**–**I** were analyzed using the Wilcoxon rank-sum test.

To ascertain if [^18^F]-FLT could be incorporated into a clinical trial that utilizes 8803, longitudinal PET imaging was performed in orthotopic glioma-bearing mice. The underlying mechanism of the association of STING pathway activation with increased signal on [^18^F]-FLT PET has been previously described (Supplemental Figure 4). When compared with PBS control mice, the [^18^F]-FLT uptake in CT-2A tumors significantly increased from baseline (i.e., pretreatment) in all mice after treatment with 8803 (Figure 6, A–D). During this time, the gliomas in control mice continued to increase in size, as measured volumetrically by MRI. In contrast, treatment with 8803 reduced the tumor volume (Figure 6E). There was no difference in the tumor proliferative rate measured by the mean percentage of Ki-67 IHC expression between the control and 8803 treatment groups, eliminating cellular turnover as a confounder for TYMP induction (Figure 6F). To simulate the imaging of epigenetic silencing of STING in the glioma, *Sting*-KO CT-2A cells were orthotopically implanted in mice and imaged with [^18^F]-FLT PET at 72 hours after 8803 treatment. The [^18^F]-FLT uptake in 8803-treated *Sting*-KO CT-2A gliomas significantly increased from baseline (i.e., pretreatment) to 72 hours, whereas there were no significant changes in the control group (Figure 6, G and H). As in parental CT-2A, *Sting*-KO CT-2A gliomas demonstrated reduced tumor volume relative to controls with 8803 treatment (Figure 6, H and I). These data indicate that [^18^F]-FLT PET imaging may be a helpful adjunct for monitoring responses in clinical studies incorporating 8803.

## Discussion

We have previously reported the efficacy of monotherapeutic 8803 across multiple preclinical models of glioblastoma, including in combination with anti–PD-1 (10). Although this would position us for implementing a first-in-human trial in the recurrent setting, additional preclinical modeling is necessary to determine how 8803 could be implemented in the up-front setting and in combination with standard-of-care radiation. Since radiation is cytotoxic to proliferating cells, including those involved in immune responses, the administration of STING agonists was delayed until after radiation completion. This type of schedule would allow radiation to induce DNA damage, antigenic shedding, and the release of DAMPs, triggering innate immunity by activating innate immune cells by TLR ligands. To mitigate the confounders of temozolomide-induced lymphopenia and immune suppression in the context of an immunotherapeutic strategy, preclinical studies were conducted using the combination of 8803 and radiation. This approach is viable for clinical trial design, as glioblastoma patients with MGMT-unmethylated tumors are unlikely to benefit from temozolomide (25). As such, many neuro-oncologists are willing to forgo the combination of radiation and temozolomide in MGMT-unmethylated glioblastoma patients after resection and refer these patients to clinical trials evaluating RT in combination with other agents.

Although it is easy to perform radiation and an intratumoral injection of STING on the same day in preclinical models, this would be a logistical challenge for both the patient and the treating radiation and surgical teams. Multiple stereotactic procedures for administering STING would also increase both patient risk and cost. As such, we modeled in mice the scenario in which, upon completion of radiation, the individual would recover for several days while the surgical team prepared for the case. This short interval after radiation would allow the immune-adjuvant effects to still be present in the patient alongside a contrast-enhancing target, as enhancement can take several weeks to months to resolve. This was the rationale for modeling the preclinical schedule. Based on our preclinical results, a 72- to 96-hour posttreatment PET evaluation would be an appropriate time to detect STING-dependent changes in a clinical trial of 8803. The 3-day interval between the completion of radiation and treatment with 8803 will also provide a reasonable logistical time for obtaining a baseline [^18^F]-FLT PET scan. STING expression is epigenetically silenced in the glioblastoma tumor cells but remains active in the immune system (3). This is not the case for immune-competent murine glioblastoma cell lines, which may overestimate responses to STING agonists since more STING is expressed in the TME. [^18^F]-FLT PET uptake can also be seen in preclinical glioma models in which the cGAS has been targeted (26). There are still therapeutic effects of 8803 in humanized murine glioma models. However, the responses are not as biologically compelling as those observed in syngeneic clonotypic immune-competent models (10). We previously showed that STING agonists can induce marked radiographic responses in canines with glioblastoma (10), but the STING promoter methylation status in these tumors remains unknown. As such, we modeled this epigenetic silencing in murine glioblastoma CT-2A using CRISPR and then evaluated the therapeutic effect of the combination using a clinically feasible schema. Despite the absence of STING in glioma tumor cells, the combination of radiation and 8803 still demonstrated biologically significant treatment responses (Figure 5D), implicating the role of STING in the immune system in mediating these effects, including in combination with radiation. The observation that the combination of 8803 with RT had a lower frequency of effector responses relative to monotherapeutic 8803 may be a function of kinetics, as the combination received a week of immune-activating RT before analysis. Alternatively, T cells highly activated by the combination therapy could undergo apoptosis (27).

During treatment, scRNA-sequencing analysis from the gliomas revealed that the combination of RT + 8803 increased the frequency of the Inflam-TAM(2) population distinguished by the expression of Nos2, which encodes nitric oxide synthase that can mediate cytotoxic action against tumor cells (28). NO modulates tumor DNA repair mechanisms by upregulating p53, PARP, and DNA-PK. The therapeutic effect of 8803, RT, or the combination of RT + 8803 was not ablated in the NOS2-KO background mice, indicating that this pathway is not a major contributor to therapeutic activity. The therapeutic efficacy of the RT in the NOS2-KO background was enhanced when compared with the MS of the C57BL/6J background, consistent with the previously documented role of NOS as a radiation sensitizer (29, 30). Although the Inflam-TAM(2) population could serve as a response biomarker, this cellular population is not a key mediator of the RT + 8803 therapeutic response based on the NOS2-KO background outcomes. Instead, the combinatorial additive effect is due to the well-documented 8803-induced immune-mediated cytotoxicity and the direct cytotoxic effects of radiation. STING regulates transcriptional programs that control the generation of ROS. More specifically, the loss of STING has been shown to alter ROS homeostasis and reduce DNA damage, thereby inducing therapeutic resistance to radiation (5). The pharmacological activation of STING enhances the effects of ionizing radiation, thereby providing a rationale for the combination. Although stromal STING expression is required for the maximal response to high-dose single-fraction RT through interactions with CD8^+^ T cells, the contribution of tumor-expressed STING has not been determined (31).

Despite decades of use, including complex genetically engineered murine models of glioblastoma, preclinical glioma models have not predicted clinical trial outcomes spanning a wide variety of therapeutic classes. Unfortunately, these models do not fully recapitulate the heterogeneity, invasion, size, complexity, genetic, and immunological aspects that are reflective of human glioblastoma. In this study, we modeled the epigenetic silencing of STING in CT-2A to simulate the scenario in human glioblastoma and still found a therapeutic response with the combination of RT and 8803. Differences in therapeutic response between preclinical glioma models (CT-2A vs. QPP8v) may be a function of stemness (32), which may require consideration as a response biomarker. Ultimately, only human clinical studies will resolve whether this combination is meaningful, but the preclinical study has provided the logistical framework. Limitations of the current study include the lack of identification of a clear response biomarker(s), elucidation of the mechanism of therapeutic resistance, and whether radiation in combination with 8803 blunts immunological memory, toward which future dedicated studies will need to be directed. Based on the rapidly emerging literature, multiple mechanisms of treatment resistance are likely operational and will be cancer lineage specific and based on factors such as the degree of myeloid infiltration and the epigenetic silencing of STING (3). Therapeutic resistance to STING agonists can also be acquired through mutations and alterations of STING isoforms that render them insensitive to stimulation (33). Pharmacological modulation using low pulsed dosing with STING agonists is critical for boosting T cell and antigen-presenting responses without inducing cell cycle–limiting and immunosuppressive feedback due to the effects of excess IFN signaling and high levels of neutrophil activation. Careful dose titration of 8803 will be required when administered immediately after radiation, since higher doses of 8803 may not yield greater therapeutic benefit as was noted in the CT-2A preclinical model.

Our current study demonstrates that the absence of tumor cell STING expression does not negate the response to RT and 8803, and it still generates a therapeutic signal sufficiently compelling to warrant consideration in clinical trials. Patients with greater STING expression may have a greater therapeutic response, which could be quantified based on STING promoter methylation status (3). However, there is no definitive predictive radiation response biomarker on a Clinical Laboratory Improvement Amendments–approved (CLIA-approved) platform that could be used to identify glioblastoma patients more likely to respond to the combination of RT and 8803. Alternatively, since IFN is downstream from STING activation, the CLIA-certified platform IFN signature (34) could be measured to predict the response to the combination therapy. Finally, since only a few agents achieve sufficient brain concentration to mediate therapeutic activity in our current compendium, the observation that the STING agonist 8803 can open the BBB for a sustained period provides a likely new and significant avenue for future investigation into combinatorial strategies.

## Methods

### Sex as a biological variable.

Our study examined male and female animals, and similar findings are reported for both sexes.

### Cell lines.

The CT-2A (Sigma-Aldrich; SCC194) and RAW 264.7 (ATCC; TIB-71) murine cell lines were maintained in Dulbecco’s modified Eagle medium (DMEM; Corning) with 10% fetal bovine serum (FBS). The QPP8v cell line (obtained from MD Anderson, Houston, Texas, USA) was maintained using NeuroCult Basal Medium and Supplements kit (Stem Cell Technologies, catalog 05702) in addition to EGF (20 ng/mL), bFGF (20 ng/mL), and B27.

### Animal models.

C57BL/6J mice (strain 000664) were purchased from Jackson Laboratories and implanted with a cranial cannula (Protech International) that is compatible with a 26-gauge 1700 series 10 μL gas-tight Hamilton syringe (catalog 80075) loaded onto a multi-injector system with an infusion rate of 0.5 μL/min (PHD 2000 syringe pump catalog 70-2000, Harvard Apparatus). *Nos2^tm1Lau^* targeted mutation KO mice in the C57BL/6J background were purchased from Jackson Laboratories and have been previously described (35). To induce intracerebral tumors in C57BL/6J mice, glioma cells were collected in the logarithmic growth phase using trypsin EDTA, washed, and resuspended in PBS. The intracerebral tumorigenic dose was 1 × 10^5^ for CT-2A, 1.5 × 10^5^ for CT-2A STING KO, and 2 × 10^5^ for QPP8v, in a total volume of 2.5 μL. Male and female mice were then randomly assigned to vehicle control or treatment groups. The mice were observed daily for survival recording and were compassionately euthanized upon signs of neurological deficit (lethargy, hypothermia, failure to ambulate, lack of feeding, body condition score <2.0, or loss of >20% body weight).

### Generation of STING knockout in CT-2A.

Single-gene-knockout clones were generated in CT-2A mouse glioma cells using the lentiCRISPR v2-Blast vector, which is from Mohan Babu (Canada, Addgene plasmid 83480; RRID: Addgene_83480). The protocol for guide cloning and the generation of the virus has been previously described (23). The guide sequence for STING KO is GATGATCCTTTGGGTGGCAA, and the nontargeting control is CACCAATATTTGGCTCGGCTGCGC. The STING KO and control clones were selected using blasticidin from Sigma (5 μg/mL). The STING KO was confirmed on Western blot using the anti-STING (D1V5L) rabbit mAb 50494 from Cell Signaling Technology, used at a dilution of 1:1,000. The β-actin (13E5) rabbit mAb 4970 was from Cell Signaling Technology, used at a dilution of 1:8,000.

### In vivo treatments.

The STING agonist 8803 was administered i.c. via cannula, as per the treatment schema, at a concentration of 2.5 or 5 μg/mouse. RT was administered daily for 5 days at 2 Gy fractions, resulting in a total dose of 10 Gy per mouse. Dexamethasone was administered via oral gavage at 10 mg/kg per mouse concurrent with 8803 for BBB opening evaluation.

### BBB permeability experiment.

C57BL/6J mice were purchased from Jackson Laboratories and divided into the following groups (*n* = 2–3/group): untreated, untreated with fluorescein, RT (fraction of 2 Gy) with fluorescein, PBS (i.c. injection of 2.5 μL 1× PBS) with fluorescein, 8803 (5 μg/mouse i.c. injection) with fluorescein, RT + PBS + fluorescein, and RT + 8803 + fluorescein. Intravenous fluorescein (NaFl; Sigma-Aldrich) was administered at a dose of 20 mg/kg diluted in 0.9% saline solution to a final injection volume of 100 μL 45 minutes before euthanasia. The RT mice were euthanized, and the brains were collected 24 hours after fraction administration. The PBS and 8803-treated groups were euthanized, and brains were collected at 2, 6, and 24 hours after i.c. drug administration. The brains were extracted and imaged using the Nikon AZ100 epifluorescence microscope (4×, FITC filter cube, 600 ms exposure time). Higher magnification images of the vessels were taken at a 150 ms exposure time. Analysis of the images was done in FIJI/ImageJ. The signals in all images were set at the same threshold, and the signal was collected.

### IHC and Luxol Fast Blue staining.

Tissues were embedded in paraffin and cut into 4 μm–thick sections on positively charged slides. Dewaxed sections were stained with hematoxylin and eosin for histological evaluation. Luxol Fast Blue-Cresyl Echt Violet stain (Thermo Fisher Scientific) was performed according to standard protocol (36). IHC staining was conducted using a STING primary antibody (Cell Signaling Technology, clone D2P2F, catalog 13647) or NRF2 primary antibody (Thermo Fisher Scientific, PA5-27882) and a poly-HRP conjugate goat anti-rabbit secondary antibody (Invitrogen, catalog B40962). IHC slides were counterstained with hematoxylin before being mounted and stored at room temperature overnight to dry before analysis.

### scRNA-sequencing analysis.

At designated time points, tumor-bearing mice were euthanized and brains collected following cardiac perfusion and chopped up using a scalpel, dissociated, and suspended using a mixture of IMDM 1× (Corning) containing 2% inactivated FBS (MilliporeSigma), collagenase (100 μg/mL), and DNase (20 U/mL). Samples were incubated for 35–40 minutes at 37°C with agitation. The tissue was filtered through a 70 μm nylon cell strainer (BD Biosciences) and then underwent centrifugation at 300*g* at 4°C. Half of the pellet representing the whole TME was resuspended in culture media and the other half in a 20 mL mixture of 5.4 mL Percoll Plus (GE Healthcare, now Cytiva) overlaid with 12 mL 1× PBS and 0.6 mL 10× PBS (Corning) for immune enrichment. The tube was centrifuged at 800*g* for 10 minutes at 4°C, with 9 acceleration and 0 deceleration. After centrifugation, the immune-enriched cell pellet was collected, washed, mixed with the whole TME samples at a ratio of 1:1, stained with trypan blue dye (MilliporeSigma), and counted using a Countess II FL automated cell counter in a Countess cell-counting chamber (Invitrogen). After library preparation, cells were sequenced using Illumina NovaSeq. Preprocessing of raw sequencing files included alignment to mm10 reference using Cell Ranger (v3.1.0). Next, ambient mRNA and doublets were removed using SoupX (v.1.3.0) and sDblFinder (v1.4.0), respectively. Then, a standard Seurat R Package workflow was applied (37). Cells were cleaned using a mitochondrial DNA threshold of 10% and a UMI range of 100 to 5,000. In addition to Seurat functions, the Harmony algorithm was used for batch correction (38). Nontumor cell clusters were annotated using 3 parallel methods: 1) comparison against known cell markers and myeloid subtypes (13, 16), 2) examination of DEGs against the Human Protein Atlas, and 3) singleR package, an automated cell assignment algorithm. Differential abundance testing was conducted using the MiloR package (39).

### NO ELISA.

RAW 264.7 cells (1 × 10^6^) were plated into 6-well plates and irradiated at 2 Gy. After 12 hours, 8803 (10 μM) was added, and then NO release was measured 12 hours following the addition of 8803. The level of nitrite (a stable metabolite of NO) in the supernatant was measured using the Griess reagent (Invitrogen, G7921) according to the manufacturer’s instructions.

### [^18^F]-FLT PET imaging.

The [^18^F]-FLT probe was obtained from the Cyclotron Facility at the University of Chicago. Glioma-bearing mice were imaged with MRI (Biospec 7 T, Bruker) and PET/CT (NanoScan, Mediso) on days 18 and 20 after i.c. cell implantation of CT-2A cells (1 × 10^5^) after being randomized into treatment groups based on tumor size. The [^18^F]-FLT probe was administered intravenously via tail vein injection, and brain images were acquired 1.5 hours after probe administration. Pretreatment PET/CT scans of each glioma-bearing mouse were followed immediately by intratumoral injection through implanted cannulas of the STING agonist IACS-8803 at a dose of 5 μg/mouse. Control mice were also scanned and treated with PBS. PET/CT examinations were then repeated (on both 8803-treated and PBS control mice) at 48 to 96 hours, based on radiotracer availability from the University of Chicago, after the 8803 treatment. PET data sets were reconstructed using the Tera-Tomo 3-dimensional reconstruction and CT images for attenuation correction. The coregistered MRI provided the soft tissue anatomical image reference for the accurate delineation of the tumor region in each mouse. VivoQuant software was used to analyze the reconstructed images. Regions of interest were drawn manually using the coregistered MRI images as the anatomical reference for accurate tumor segmentation. Tumor-specific [^18^F]-FLT uptake was quantified as a percentage of injected dose normalized to the optical nerve as reference tissue. The multiple sets of normalized data were then used for the assessment of 8803-induced tumor alterations linked to the therapeutic effect. Longitudinal tracking and regional analysis of PET-derived [^18^F]-FLT uptake maps as a function of time were possible through an advanced image-processing pipeline that relied on the mutual information from coregistered multimodality images. This method enabled tumor-specific quantitative parametric values to be obtained reflective of therapeutic cellular changes (i.e., increased STING activation through detection of treated mouse tumor PET uptake) and altered anatomical intratumoral features, such as tumor morphology and size using MRI, which were used for quantitative and statistical comparison between individuals and at different time points. The treatment injection track was visualized in MRI images to ensure treatment location. Only mice with successful intratumoral delivery were analyzed.

### CNS toxicity assessments with 8803 and radiation treatment.

To evaluate whether treatment with this combination induces an inflammatory response directed against the CNS, the mice were evaluated daily. No neurological symptoms were noted. When the mice succumbed to the tumor, the neuroaxis was stained for myelin with Luxol Fast Blue, as well as hematoxylin and eosin. On histologic examination, there was no evidence of demyelination, as reviewed by a board-certified neuropathologist. Additionally, perivascular lymphocytic infiltrates were not seen, which would implicate the induction of autoimmunity. Neuropathologic studies failed to demonstrate perivascular fibrosis or persistent demyelination consistent with subclinical autoimmunity occurring at any point during the animal’s lifespan. No evidence of induced autoimmunity was detected when the CNS was evaluated with Luxol Fast Blue.

### Statistics.

Kaplan-Meier curves were plotted to describe the overall survival starting from day 0 of tumor cell implantation and to estimate the MS time for each treatment group. A Cox proportional model was utilized by using overall survival as a response and the group as a covariate, with the primary research objective to compare the combination group with each monotherapy group. The proportional assumption was checked before model fitting. The Bonferroni method was used to adjust the *P* value significance cutoff when multiple *P* values were investigated simultaneously. The *P* values for curve comparisons were calculated using the log-rank method followed by the Bonferroni correction. *P* < 0.05 was considered significant.

### Study approval.

All in vivo mouse experiments were approved following Laboratory Animal Resources Commission Standards by the Institutional Animal Care and Use Committee and conducted according to the approved protocols IS00020425 and IS00023704 at Northwestern University.

### Data availability.

The data used to support the findings of this study are available within this article and within the Supporting Data Values file. Sequencing data are available at NCBI GEO GSE305400.

## Author contributions

Experimental design was done by ST, HN, LH, IB, and ABH. Methodology and execution were done by ST, HN, RD, HA, KJH, LH, CD, KM, and DP. Funding acquisition was done by MSL, RS, IB, and ABH. Data analysis was done by ST, HN, LH, PZ, CMH, LL, RJA, RS, RVL, TLS, DRR, SS, DMA, MAC, DP, IB, and ABH. Initial drafting and writing were done by ST and ABH. Manuscript review was done by all authors. Co–first authors are listed in descending order of their contributions to the work, with the person who contributed the most listed first. All authors approved the final manuscript.

## Funding support

This work is the result of NIH funding, in whole or in part, and is subject to the NIH Public Access Policy. Through acceptance of this federal funding, the NIH has been given a right to make the work publicly available in PubMed Central.

- NIH grants CA120813, NS120547, and P50CA221747.
- Northwestern University Research Histology and Phenotyping Laboratory, supported by the National Cancer Institute (NCI) grant P30CA060553 awarded to the Robert H. Lurie Comprehensive Cancer Center.
- Northwestern University Center for Advanced Microscopy (RRID: SCR_020996) and Small Animal Translational Imaging Core, supported by NCI P30CA060553, awarded to the Robert H. Lurie Comprehensive Cancer Center.
- 1S10-OD03221 grant awarded to the CTI core.

## Supplementary Material

Supplemental data

Unedited blot and gel images

Supporting data values

## Version 1 02/16/2026

Electronic publication
