# Supplementary material for: STING-induced blood-brain barrier opening combined with radiotherapy potentiates antitumor response in a high-grade glioma model
Source: J Clin Invest. 2026 Feb 16;136(4):e198843. doi: 10.1172/JCI198843 (PMC12904701; doi:10.1172/JCI198843)
Supplement: Supplemental data [file jci-136-198843-s019.pdf]

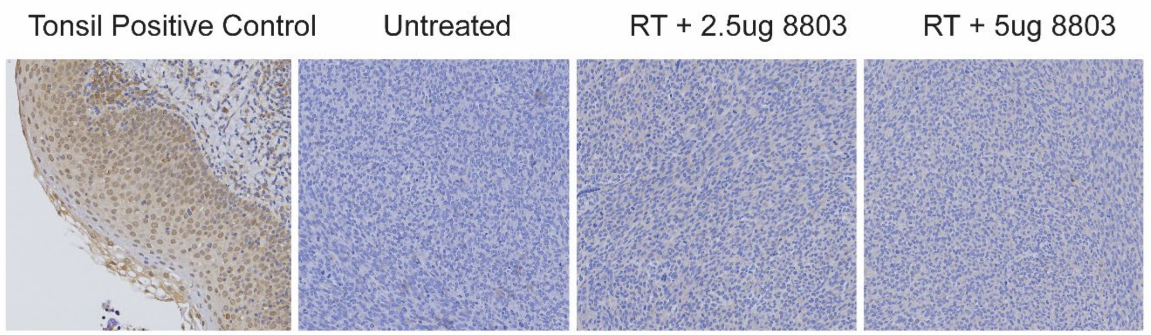

Supplementary Figure 1: Immunohistochemistry of Nrf2 in Tonsil Positive control and CT-2A brains from either untreated (PBS control), RT + 2.5ug 8803, or RT + 5ug 8803 mice. N=2-3 per treatment group.

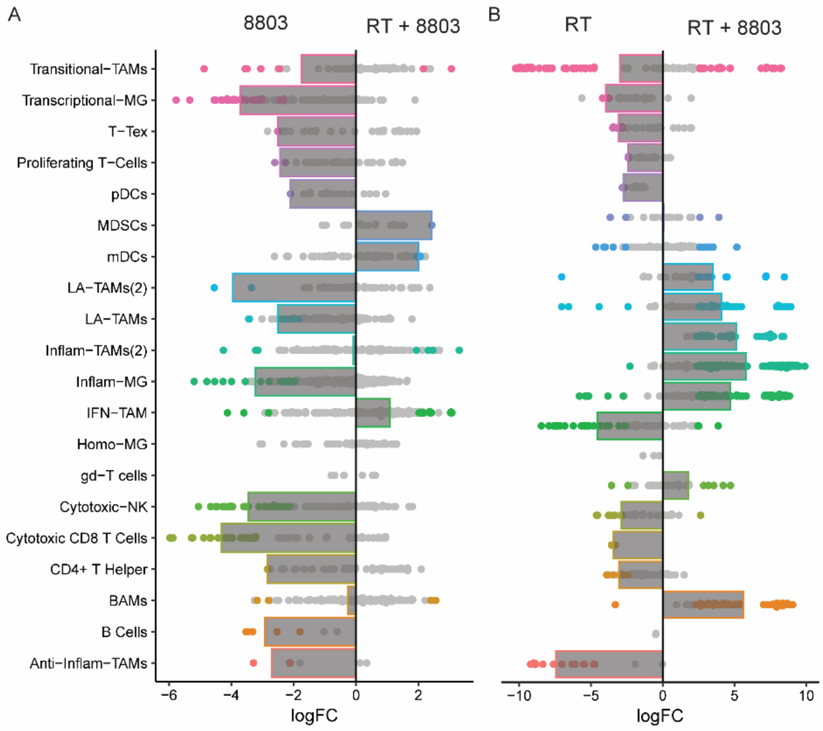

Supplementary Figure 2: Differential abundance testing of immune cell populations from scRNA between 8803 + RT vs 8803 or RT. (**A, B**) Strip plots showing the differential abundance of immune cell types in CT-2A bearing C57BL/6 mice based on treatment compared to control, log2(FC). The dot is colored when the P value < 0.05. The boxplot represents the mean of significant points for immune populations with more than 3 significant clusters. Cell types with a *P* value $\geq$ 0.05 are colored otherwise in grey. Additional comparison between experimental groups

are displayed in Fig. 2D. P value calculated using negative binomial generalized linear model with weighted false discovery rate multiple correction.

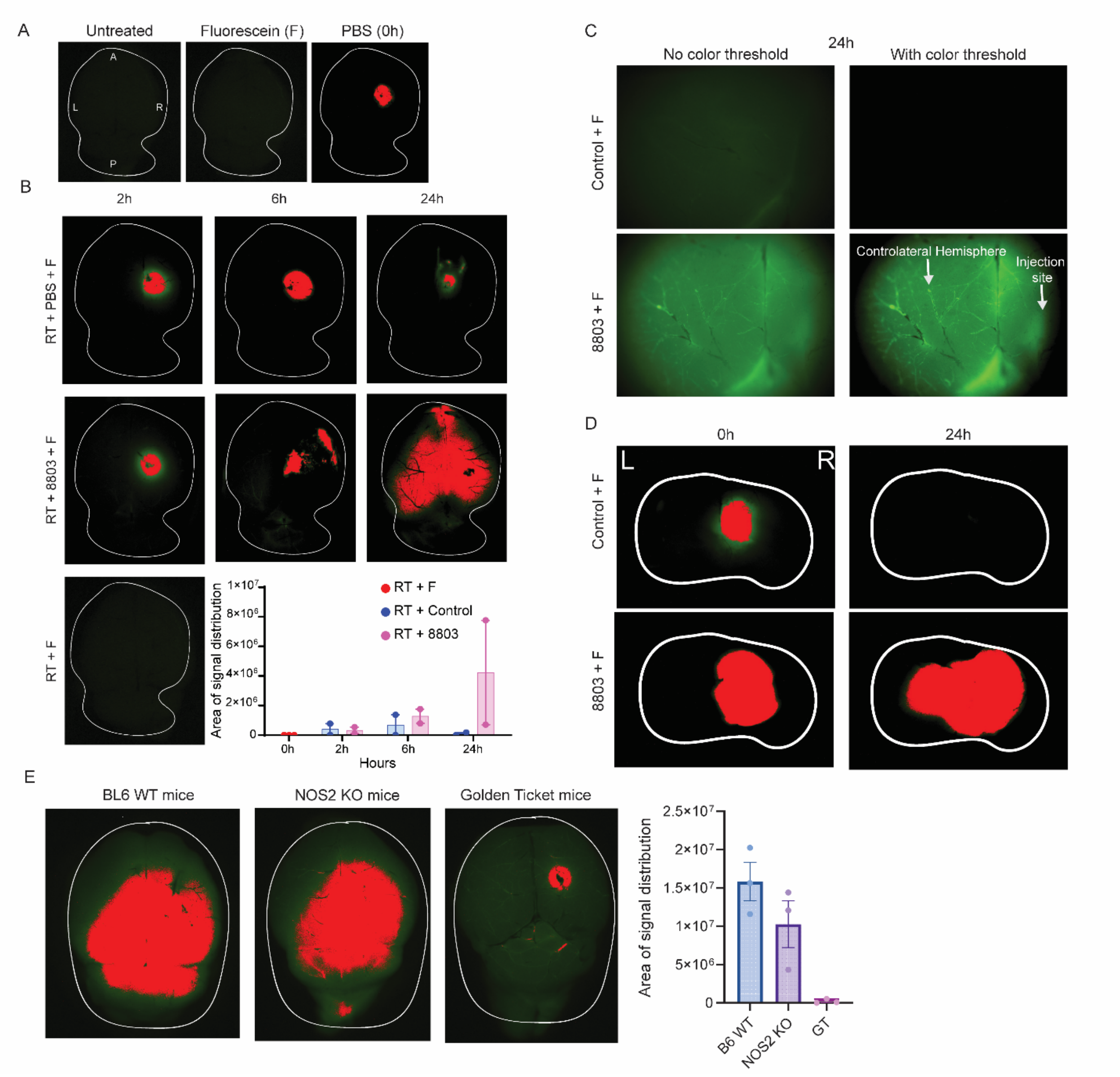

Supplementary Figure 3: **The influence of RT on 8803 triggered blood-brain barrier opening**. (**A**) Representative explanted whole brain mount images from the experimental control groups: untreated, fluorescein (F) dye only, and PBS at baseline. After fluorescein dye administration, mouse brains were collected and imaged using the Nikon epifluorescent microscope at 4x magnification. Quantitative data was generated based on intensity thresholds, eliminating noise, and then harmonizing across all images. Positive intensity data points (pixels) are shown in red, and the baseline fluorescent expression in green. The brain outline is illustrated with the white line. Brightness color adjustments were made to detect baseline green fluorescence

in the brains for illustrative purposes, which do not change the quantitative data generated based on intensity thresholds harmonized across all images. Orientation is left (L), right (R), anterior (A), and posterior (P). (**B**) Representative explanted whole brain mount images from the experimental groups: RT + PBS + F, RT + 8803 + F, and RT + F only. After fluorescein (F) dye administration, mouse brains were collected and imaged using the Nikon epifluorescent microscope at 4x magnification. Quantitative data was generated based on intensity thresholds, eliminating noise, and then harmonizing across all images. Positive intensity data points (pixels) are shown in red, and the baseline fluorescent expression in green. The brain outline is illustrated with the white line. Brightness color adjustments were made to detect baseline green fluorescence of the brains for illustrative purposes. This does not alter the quantitative data generated based on the intensity thresholds harmonized across all images. The signal area as measured by square microns was quantified longitudinally at baseline (0), 2, 6, and 24 hours. (**C**) Representative higher 4x magnification images of blood vessels taken from PBS + F and 8803 + F experimental groups at 24 hours. Exposure time at 150ms. (**D**) Coronal sections of the brains taken from experimental groups: PBS + F and 8803 + F at 0 and 24 hours. Left (L) and Right (R) are designated. Animal group sizes are n=2-4 per group and time point. (**E**) Representative explanted whole brain mount images 24 hours following 8803 and dexamethasone injection in C57BL/6, Goldenticket (STING KO) and NOS2 KO backgrounds. After fluorescein dye administration, mouse brains were collected and imaged using the Nikon epifluorescent microscope at 4x magnification. Quantitative data was generated based on intensity thresholds, eliminating noise, and then harmonizing across all images. Positive intensity data points (pixels) are shown in red, and the baseline fluorescent expression in green. The brain outline is illustrated with the white line. Brightness color adjustments were made to detect baseline green fluorescence of the brains for illustrative purposes. This does not alter the quantitative data generated based on the intensity thresholds harmonized across all images.

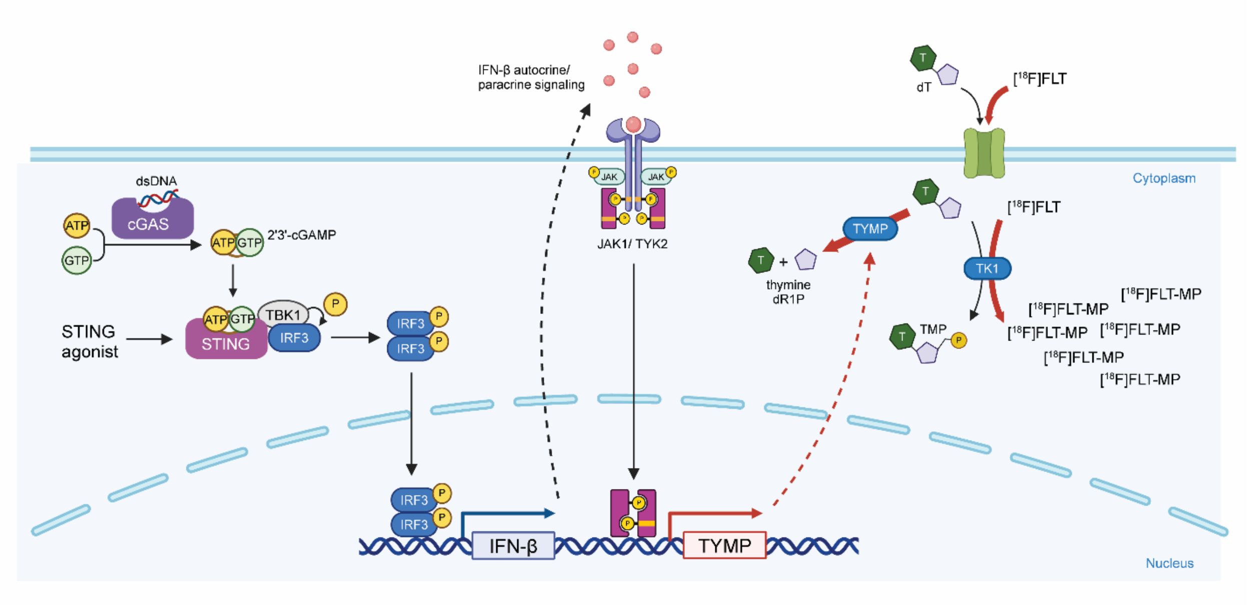

Supplementary Figure 4: Schema showing the cellular metabolism of the [^{18}F]-FLT PET tracer after STING pathway activation as summarized from (40). Interferon signaling induces cellular nucleotide metabolism through the transcriptional induction of thymidine phosphorylase (TYMP). TYMP catalyzes the first step in the catabolism of thymidine, which competitively inhibits the accumulation of the nucleoside analog PET probe 3'-deoxy-3'-[^{18}F]fluorothymidine ([^{18}F]FLT).
