# Supplementary figures and images for: STING-induced blood-brain barrier opening combined with radiotherapy potentiates antitumor response in a high-grade glioma model
Source: J Clin Invest. 2026 Feb 16;136(4):e198843. doi: 10.1172/JCI198843 (PMC12904701; doi:10.1172/JCI198843)

Full unedited gel for Figure 5A

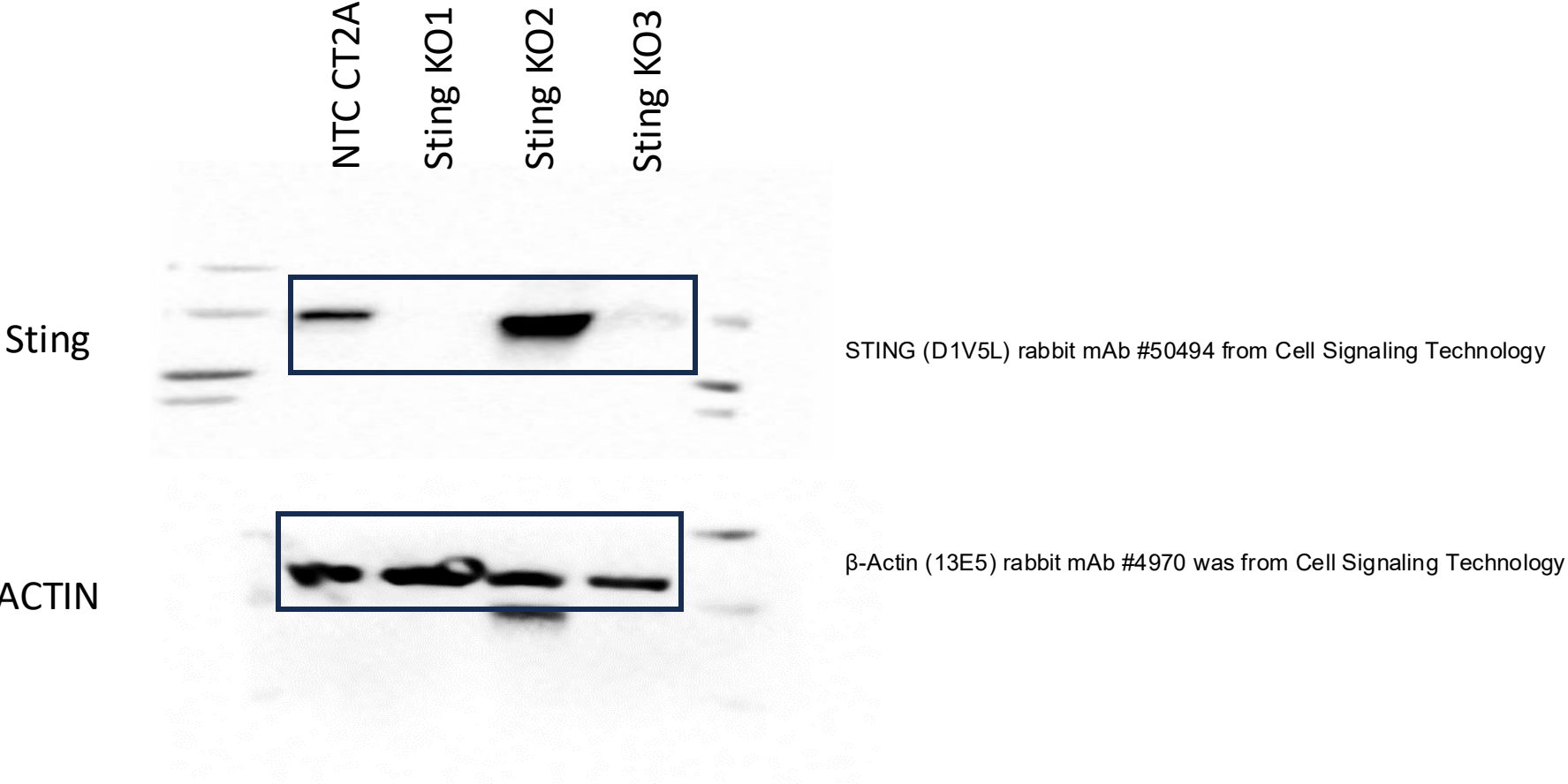

Supplement: Unedited blot and gel images [file jci-136-198843-s020.pdf]
